# Chatbot -assisted self-assessment (CASA): Co-designing an AI -powered behaviour change intervention for ethnic minorities

**DOI:** 10.1371/journal.pdig.0000724

**Published:** 2025-02-13

**Authors:** Tom Nadarzynski, Nicky Knights, Deborah Husbands, Cynthia Graham, Carrie D. Llewellyn, Tom Buchanan, Ian Montgomery, Alejandra Soruco Rodriguez, Chimeremumma Ogueri, Nidhi Singh, Evan Rouse, Olabisi Oyebode, Ankit Das, Grace Paydon, Gurpreet Lall, Anathoth Bulukungu, Nur Yanyali, Alexandra Stefan, Damien Ridge

**Affiliations:** 1 School of Social Sciences, University of Westminster, London, United Kingdom; 2 Kinsey Institute, Indiana University, Bloomington, Indiana, United States of America; 3 Brighton and Sussex Medical School, University of Sussex, Brighton, United Kingdom; 4 Positive East, London, United Kingdom; Weill Cornell Medicine, UNITED STATES OF AMERICA

## Abstract

**Background:**

The digitalisation of healthcare has provided new ways to address disparities in sexual health outcomes that particularly affect ethnic and sexual minorities. Conversational artificial intelligence (AI) chatbots can provide personalised health education and refer users for appropriate medical consultations. We aimed to explore design principles of a chatbot-assisted culturally sensitive self-assessment intervention based on the disclosure of health-related information.

**Methods:**

In 2022, an online survey was conducted among an ethnically diverse UK sample (N = 1,287) to identify the level and type of health-related information disclosure to sexual health chatbots, and reactions to chatbots’ risk appraisal. Follow-up interviews (N = 41) further explored perceptions of chatbot-led health assessment to identify aspects related to acceptability and utilisation. Datasets were analysed using one-way ANOVAs, linear regression, and thematic analysis.

**Results:**

Participants had neutral-to-positive attitudes towards chatbots and were comfortable disclosing demographic and sensitive health information. Chatbot awareness, previous experience and positive attitudes towards chatbots predicted information disclosure. Qualitatively, four main themes were identified: “Chatbot as an artificial health advisor”, “Disclosing information to a chatbot”, “Ways to facilitate trust and disclosure”, and “Acting on self-assessment”.

**Conclusion:**

Chatbots were acceptable for health self-assessment among this sample of ethnically diverse individuals. Most users reported being comfortable disclosing sensitive and personal information, but user anonymity is key to engagement with chatbots. As this technology becomes more advanced and widely available, chatbots could potentially become supplementary tools for health education and screening eligibility assessment. Future research is needed to establish their impact on screening uptake and access to health services among minoritised communities.

## Author summary

This study explores how chatbots can help people assess their health and access medical advice, particularly focusing on ethnically diverse communities. Researchers surveyed 1,287 participants and conducted 41 follow-up interviews to understand attitudes towards using chatbots for health-related conversations. Most participants felt comfortable sharing sensitive and demographic information with chatbots, especially when anonymity was guaranteed. The study found that people’s willingness to share information depended more on their previous positive experiences with chatbots than on factors such as age, gender, or ethnicity. Participants valued chatbots for being accessible, non-judgmental, and private, making them especially useful for discussing sensitive topics like sexual health. However, concerns were raised about chatbots lacking human empathy and being unable to handle complex emotional issues. To improve trust, users recommended that chatbots provide clear explanations about data security, use simple and inclusive language, and offer translations in multiple languages. They also suggested that chatbots should provide clear steps for follow-up actions, such as booking appointments or ordering home testing kits. The findings suggest chatbots could supplement healthcare services by promoting education and screening, particularly for marginalised groups. Future research is needed to assess their long-term impact on improving health outcomes and addressing health inequalities.

## Introduction

Advancements in healthcare automation and artificial intelligence (AI) offer significant potential for improving public health outcomes. The use of AI in medicine has grown rapidly and now includes applications in drug development, disease diagnosis, health monitoring, digital consultations, and personalised treatment [1]. With the help of machine learning, deep learning, and natural language processing, medical algorithms have emerged, potentially allowing for more effective disease control using complex mathematical models [2–4]. AI can also improve disease surveillance, detect abnormalities in screening tests such as mammography or cervical cytology, and provide personalised health advice based on individual risk profiles and behavioural patterns [5].

Patient-facing AI systems such as chatbots or virtual agents that imitate human-to-human conversations have been shown particularly useful in health promotion and education [6]. Chatbots are capable of using natural language processing to recognise questions and respond with pre-determined clinically validated answers. They can also use behavioural algorithms as decision-making tools, symptom checkers or online triage systems. Unlike static websites, often rich in visual content, chatbots act as a message exchange platform, with their language, terminology and phraseology being key to their successful design and implementation. Although their effectiveness and safety need to be established through high-quality evaluations, chatbots could potentially contribute to improved knowledge about diseases, increased awareness of specialist healthcare services, such as vaccination or screening, and influence health beliefs, attitudes and perceptions, having an impact on motivation, health behaviours and subsequent healthcare utilisation [7]. To date, public health chatbots have been used for treatment and screening support, counselling and mental health support, behaviour change, self-management and improved health literacy [8]. Recently, more modern chatbots based on large language models, such as ChatGPT, have the potential to transform healthcare delivery and influence individual knowledge and behaviours. The role of healthcare and psychologist researchers involved in the design of health chatbots is to understand their mechanisms of action, optimise their articulacy and fluency as well as determine their influence on behaviours and cognition.

Chatbots could benefit people from marginalised social groups including gender and sexual minorities, as well as ethnic minorities that are disproportionally affected by poor health [9,10]. For example, in the context of sexual health, stigma, discrimination and prejudice have a substantial impact on the awareness and knowledge of sexually transmitted infections (STIs), sexual health screening and subsequent access to relevant healthcare services (e.g., HIV pre-exposure prophylaxis) [11–13]. Owing to embarrassment or shame, ethnic minorities are less likely to disclose sexual health-related information such as sexual orientation to healthcare professionals [14]. Consequently, they might not be offered STI/HIV testing, resulting in a missed opportunity for diagnosis, treatment and infection control. Chatbot-assisted interventions based on self-assessment may reduce these barriers and promote STI self-testing, potentially contributing to a reduction in health inequalities. Previous research has indicated a higher frequency of chatbot-led consultations about STIs, compared with hospital consultations [15] suggesting an appetite among the public for efficient, safe and reliable chatbot intervention in sexual health.

There are several potential psychological processes related to chatbot-assisted interventions. Multiple behaviour change techniques [16] could be applied within chatbots to facilitate the adoption of healthy behaviours and habits. For example, chatbots could help with goal setting by allowing users to set clear and measurable goals and work towards achieving them [17]. They can offer self-monitoring, encouraging users to track their health behaviours, increase their awareness of health-impairing behaviours and help them identify areas for improvement [18]. Chatbots could offer personalised feedback based on individual self-assessment, helping users better understand their health and motivating them to make positive changes [19]. They could also provide social support by offering encouragement, sharing success stories and connecting users with others working on similar health goals [20]. There is also a potential for chatbots to tailor messages to an individual’s unique needs and preferences, as well as engage in problem-solving to overcome barriers to healthy behaviours [21]. In addition to these techniques, the psychological effect of self-disclosure to chatbots per se may motivate users to engage in healthy behaviours by sharing personal information about themselves [22]. Studies have shown that self-disclosure to health chatbots has positively affected user attitudes towards these types of interventions [23]. Two meta-analyses also demonstrated ‘a question-behaviour effect’ in which the act of asking personal and health-related questions has a small but significant impact on behaviour change [24,25]. Therefore, conversational AI systems such as health chatbots that ask health-related questions and incorporate behaviour change techniques may result in desired health outcomes.

In this study, we aimed to identify how to design an effective and culturally sensitive self-assessment intervention based on the disclosure of health-related information to a chatbot. In particular, we wanted to identify the level and type of information that people from ethnic minorities were willing to disclose to health chatbots. Our objective was to understand what chatbot features were acceptable to a diverse group of users and to identify factors that should be considered in the design of chatbot-assisted self-assessments (CASA), particularly in the context of sexual health screening behaviours.

## Method

### Design

We employed a mixed methods design, combining a cross-sectional anonymous online survey and follow-up interviews with a subset of participants. We assessed the comfort of disclosing health-related information to chatbots to inform the development of future CASA interventions. The study was approved by the University of Westminster Research Ethics Committee (ref: ETH2122-0524/0561).

### Participants

We aimed to recruit a diverse and representative sample, including individuals from religious and ethnic minorities, sexual and gender minority groups, and those with disabilities or chronic health conditions. The focus was on capturing the perspectives of “seldom heard” populations that are underrepresented in research. The target sample size was approximately 1,500 individuals over the age of 18 who reside in the UK, in order to align the results with the context of the UK National Health Service. Participants under the age of 18 and those residing outside the UK were excluded from the analysis.

### Recruitment

Recruitment took place from January to August 2022 using a combination of community-based and online strategies to obtain a diverse sample. A community engagement approach involved contacting over 30 UK-based third-sector organisations with a request to circulate the study advert. We invited them to express views about automated online platforms for health advice, containing a URL and QR code that directed them to the online survey page on Qualtrics. Leafleting and poster-based approaches were also used within community settings, such as regions of London with higher representation of people from ethnic minorities (i.e., shops, community centres, and religious groups). A professional leafleting agency was also employed to distribute the study adverts in Manchester, especially in postcodes with higher ethnic diversity. Our partner organisation Positive East distributed leaflets amongst their networks across East London and via their sexual health and HIV testing outreach activities.

The advert was also distributed in university and higher education settings (i.e., university campuses, student union groups, and student association social media accounts). Similarly, paid advertisements via Facebook, Instagram and Prolific, and free adverts on the X Platform (formerly Twitter), were utilised to reach various groups such as those living in non-urban areas, sexual minority groups or specific social groups (i.e., trans and gender diverse). Face-to-face meetings with interested organisations were arranged. The study was also promoted via radio broadcasts and in publications directed towards specific ethnic minorities, such as Latin Americans living in the UK. Lastly, a snowball sampling approach was utilised with community champions and university students representative of different ethnic minorities to further distribute the study advert within their networks. Due to such varied recruitment methods, it was not possible to record the number of eligible participants or responses related to each recruitment approach. Three optional prize draws of £100 each were offered as an incentive for the completion of the survey.

### Procedure

Participants who clicked on the study advertisement link were taken to an online survey on Qualtrics, which presented an information page and an electronic consent form. The survey consisted of demographic and attitudinal questions, such as opinions on health and sexual health chatbots, comfort level in disclosing information to chatbots, and expected reactions to chatbot recommendations for health screening. Additionally, to better understand participants’ risk of STIs, the survey included behavioural questions related to sexual health, such as the number of sexual partners and condom usage. For those unfamiliar with chatbots, a description of a health chatbot was provided in the supplementary material (S1 File). Upon completion of the survey, participants were redirected to a debriefing page that included contact information for sexual health organisations and a separate online form where they could opt-in to participate in the optional prize draw, as well as follow-up interviews by providing their email addresses.

The sample for the interview was purposefully selected to ensure diversity in terms of age, gender, ethnicity, and sexual orientation. Participants were selected based on a maximum variation sampling framework, based on their ethnicity, and were scheduled on a first-come, first-served basis. The interviews were conducted either in person at the University of Westminster or online through MS Teams, according to participant choice. The semi-structured interviews explored participants’ views on health chatbots using a topic schedule ( S1 File). During the interview, a demonstration of an existing sexual health chatbot – Pat (www.positiveeast.org.uk/chattopat) was conducted to allow them to interact with the technology. The participants were then asked about the different features of the intervention and their willingness to disclose health-related information. The 60-minute interviews were recorded and transcribed verbatim. Participants received a voucher worth £45 as a token of appreciation for their time.

### Measures

The questionnaire was designed by the research team with an expertise in chatbot acceptability. The survey was consulted with our public and patient involvement group to validate its readability and comprehension. The questionnaire was also piloted on 10 members of ethnic minority groups where ‘think aloud’ technique was used to validate each question. The final survey consisted of six sections. The first section collected demographic information, including age, language spoken at home, gender identity, assigned sex at birth, presence of disability or chronic illness, education, and ethnicity [26]. These questions were taken from a set recommended by the UK Office for National Statistics [27]. Participants were then informed about health chatbots and asked about their experience with this technology, their awareness of chatbots and the likelihood of using one if available, to gauge acceptability. Next, a 10-item scale was presented to assess participants’ attitudes towards health chatbots. Sample items included statements such as “*I would use a health chatbot if my doctor recommended it*” and “*Talking to a health chatbot would be frustrating*”. Participants were asked to rate their agreement with these statements using a 5-point scale, ranging from “*Strongly agree*” to “*Strongly disagree*”. Afterwards, participants were asked about the frequency of their internet searches for sexual health information and their comfort level in discussing sexual health with healthcare professionals, using a 5-point scale ranging from “*Very comfortable*” to “*Very uncomfortable*.” They were then asked to rate their risk of getting STI compared to others of the same age, using a 5-point scale from “*Much below average*” to “*Much above average*.” The survey also included questions about the gender and number of sexual partners, to determine participants’ risk of STIs. A set of 21 items was used to gauge the comfort level of participants in disclosing specific information such as their name, ethnicity of sexual partners, and symptoms of a sexually transmitted infection. The five response options ranged from “*Very comfortable*” to “*Very uncomfortable*”. A higher score indicated a greater discomfort in disclosing information to the chatbot. Participants were then presented with four scenarios in which a chatbot made testing recommendations based on the answers they provided, such as “*Based on your answers, you are at a higher/average/lower risk of STIs compared to people your age*”. Participants were asked to choose one of seven anticipated responses, such as visiting a sexual health clinic, ordering a test, or taking no action. The final part of the survey included a 14-item scale that assessed participants’ attitudes towards sexual health chatbots. The items, such as “*A sexual health chatbot could help me find information about STIs and HIV*” and “*I would not trust information provided by a chatbot about my sexual health*”, were presented with five response options ranging from “*Strongly agree*” to “*Strongly disagree*”. The last question in the survey gauged the acceptability of a sexual health chatbot by asking participants, “*If a sexual health chatbot was available today, how likely would you be to use it?*”

The follow-up interviews searched deeper into participants’ reasons for sharing information and their attitudes towards health chatbots. Participants were asked to discuss ways to better establish trust in the technology, and increase engagement with the CASA intervention. They were also asked to voice any concerns they had about chatbots and their reactions to personalised health recommendations. The purpose of these discussions was to deepen our understanding of how to improve CASA as a potential psychological support tool for users making health-related decisions.

### Data analysis

The research team conducted a thorough examination of the quantitative data to ensure accuracy and reliability. Inclusion criteria were checked and cases with no variation in item responses were removed. Descriptive statistics were used to understand the distribution of data and to determine if parametric assumptions were met. The 21 items related to participants’ comfort in disclosing information to chatbots were explored using Principal Components Analysis and Varimax rotation, which revealed three distinct sets of items: “*sensitive health information”*, “*demographic information*”, and “*personally identifiable information*”. Mean scores for each set were calculated as dependent variables for further analysis. A one-way ANOVA was performed to identify differences in disclosure between White British participants and those from minoritised ethnic groups. Correlation coefficients were calculated to understand associations between variables and linear regression models were used to identify predictors of disclosure for the three sets of items. Categorical predictors were analysed using t-tests or non-parametric equivalents, and only predictors with a significance level of *p* = .01 or higher were included in the models. ANOVA models were used to compare categorical groups or their interactions as appropriate.

The qualitative data were analysed by three researchers (TN, NK and DR) using Braun and Clarke’s thematic analysis approach [28]. The process involved familiarising with the interview recordings and data, and then transcribing all interviews into NVivo. The transcripts were coded using a pre-determined coding schedule and individually reviewed. Coding reports were generated containing relevant quotes grouped according to specific codes. The codes were then grouped and regrouped by two researchers to identify themes and subthemes, which were then discussed and organised into clear and consistent categories by the three researchers. Finally, the final themes and quotes were reviewed and agreed upon to ensure clarity and uniformity.

### Public and patient involvement

This research involved engaging a public and patient involvement group at critical points, especially before finalising the research results. This involvement guaranteed that the analysis reflected real-world viewpoints and addressed issues related to the co-design of an AI intervention for people from disadvantaged social groups. It consisted of six members from minority ethnic groups, who actively assessed and endorsed the study’s discoveries during seven organized gatherings. They contributed to various aspects of the research, from analysing the data to enhancing the final concept of CASA. Their perspectives played a vital role in refining the proposed chatbot intervention, so that it can be optimised for people from ethnic minorities.

## Results

### Sample characteristics

The study included a sample of 1,287 participants (Table 1), of which 57.7% were women, and 59.7% were between the ages of 20 and 29. The sample was diverse in terms of ethnicity, with 28% identifying as White, 28.3% as Asian, 25.7% as Black, 8.5% as Mixed race, and 9.5% as other ethnicities. 78% of the participants were native English speakers, and 64% had a university degree or higher. 20.3% reported having a physical or mental health condition lasting more than a year, 86% identified as heterosexual and 21% had not been sexually active for the past 12 months. 72.6% considered themselves to have a low risk of STIs, and 65.2% were comfortable discussing sexual health with healthcare professionals. 76% were aware of chatbots, and 70% had previous experience with chatbots, with 26% having used them for healthcare. Participants had mostly neutral to positive attitudes towards health chatbots and sexual health chatbots, and over half of the participants expressed willingness to use chatbots for healthcare and sexual health.

**Table 1:** Sample characteristics.

Variable	Total number (%) [mean; SD]	Variable	Total number (%) [mean; SD]
**Demographic variables**		**Chatbot-related variables**	
Gender		Ever heard of a chatbot	
Male	499 (38.8)	Yes	984 (76.5)
Female	768 (59.7)	No	282 (21.9)
Non-binary	13 (1.0)	Not sure	21 (1.6)
Other/Prefer not to say	7 (0.5)	Ever used a chatbot	
Gender identity different than at birth		Yes	896 (69.6)
Yes	29 (2.2)	No	363 (28.2)
No	1,257 (97.8)	Not sure	28 (2.2)
Age in years	[30.6 (10.1)]	Ever used a chatbot for healthcare	
18-19	12 (<1)	Yes	334 (26.0)
20-29	768 (59.7)	No	887 (69.0)
30-39	269 (20.9)	Not sure	64 (5.0)
40-49	152 (11.8)	Likelihood of using chatbot for healthcare	
50+	86 (6.5)	Unlikely	269 (21.7)
Ethnicity		Not sure	328 (25.5)
White British	200 (15.7)	Likely	690 (52.8)
White Irish	9 (<1)	Likelihood of using chatbot for sexual healthcare	
White Roma or Irish Traveller	5 (<1)	Unlikely	259 (22.9)
White Other	144 (11.3)	Not sure	251 (19.5)
Mixed White and Black Caribbean	26 (2.0)	Likely	620 (57.6)
Mixed White and Black African	33 (2.6)		
Mixed Other	49 (3.8)	**Sexual health-related variables**	
Asian Indian	175 (13.7)	Preference of sexual partners	
Asian Pakistani	54 (4.2)	Opposite gender	1,009 (86.1)
Asian Bangladeshi	46 (3.6)	Same-sex male	64 (5.5)
Asian Chinese	41 (3.2)	Same-sex female	32 (2.7)
Asian Other	44 (3.5)	Both genders	61 (5.2)
Black African	257 (20.2)	Non-binary/diverse	6 (<1)
Black Caribbean	43 (3.4)	Number of sex partners in 12 months	
Black Other	27 (2.1)	Zero	269 (20.9)
Arab/Middle Eastern	40 (3.1)	One	564 (43.8)
Latin American	43 (3.4)	Two or more	404 (31.3)
Any Other	37 (2.9)	Not disclosed	50 (4)
Language spoken at home		Risk of STI compared to others	
English	966 (78.7)	Below average	898 (72.6)
Other	262 (21.3)	Average	250 (19.4)
Education		Above average	139 (8.0)
No formal qualifications	17 (1.3)	Comfort discussing sexual health with HCP	
5 or less GCSE/O level or equivalent	86 (6.7)	Comfortable	803 (65.2)
2 or more A levels or equivalent	191 (14.8)	Neutral	178 (13.8)
Higher education below degree level	160 (12.5)	Uncomfortable	304 (21.0)
Degree or equivalent	472 (36.8)		
Postgraduate degree or equivalent	338 (26.4)		
Other	17 (1.3)	**Attitudes towards chatbots**	
Mental or physical health condition > 12 months		Attitudes towards health chatbots	[2.5; (0.60)]
Yes	261 (20.3)	Attitudes towards sexual health chatbots	[2.5; (0.60)]
No	989 (80.7)		
**Information disclosure to chatbots**			
Sensitive health information	[2.26; (0.96)]		
Demographic information	[1.66; (0.89)]		
Personally identifiable information	[2.56; (1.29)]		

HCP = healthcare professional; STI = sexually transmitted infection; SD = standard deviation

### Disclosure of information to chatbots

Most participants were “*somewhat comfortable*” disclosing information to chatbots (S1 Table). A within-subjects one-way ANOVA indicated that participants were more comfortable disclosing sensitive health information and demographic information than personally identifiable information F = 66.51, *p* = <.001. There was no significant difference across ethnic groups (S2 Table) in the level of discomfort disclosing sensitive private information F = (4, 384.62) = 1.47, *p = .*21; demographic information F = (4, 384.03) = 1.56, *p =* .18; or personally identifiable information F = (4, 376.58) = 2.07, *p = .*08).

The overall model for predictors of discomfort disclosing sensitive health information was significant R² = 0.43, F (9, 1,112) = 94.19, *p < .*001. Awareness of chatbots (β = .157, *p = .*04*),* previous experience with chatbots (β = .176, *p = .*01*),* comfort discussing sexual health with HCPs (β = .166, *p <* 001*),* sexual health chatbot acceptability (β = −.06, *p* = .02), and attitudes towards sexual health chatbots (β = .869, *p <* 001) predicted degree of discomfort disclosing sensitive health information.

The overall model for predictors of discomfort disclosing demographic information was significant R² = .26, F (5, 1,109) = 78.55, *p < .*001. Male gender (β = −.153, *p <* 001), previous experience with chatbots (β = .179, *p =* .01), attitudes towards health chatbot (β = .133, *p = .*01*),* and attitudes towards sexual health chatbot (β = .638, *p <* 001) predicted degree of discomfort disclosing demographic information.

The overall model for predictors of discomfort disclosing personally identifiable information was significant R² = .157, F (5, 1,121) = 41.73, *p <* .001. Attitudes towards sexual health chatbots (β = .738, *p <* 001) were the only predictor of discomfort in disclosing personally identifiable information.

### Reaction to chatbot risk advice

There were various reactions to chatbot risk estimations (Table 2). In the ‘lower risk of STIs’ scenario, most participants (42%) would do nothing or order a home testing kit (20%). In the ‘average risk of STIs’ scenario, most participants would order a home testing kit (26%), call a sexual health clinic (20%), or visit a sexual health clinic (19%). In the ‘higher risk of STIs’ scenario, most participants would visit a sexual health clinic (51%), call a sexual health clinic (17%), or order a home testing kit (14%). In the ‘too early to test’ scenario, most participants would visit a sexual health clinic (32%), call a sexual health clinic (28%), or order a home testing kit (13%). Less than 5% of participants would either talk to a chatbot again or a friend/family member across all scenarios.

**Table 2:** Anticipated reactions to chatbot risk appraisal of sexually transmitted infections.

Anticipated reaction	At lower risk N (%)	At average risk N (%)	At higher risk N (%)	Too early to test N (%)
Visit a sexual health clinic	134 (10.4)	240 (18.6)	657 (51.0)	406 (31.5)
Call sexual health clinic	121 (9.4)	260 (20.2)	219 (17.0)	366 (28.4)
Order a home testing kit	259 (20.1)	335 (26.6)	177 (13.8)	169 (13.1)
Do nothing	545 (42.3)	229 (17.8)	55 (4.3)	106 (8.2)
Talk to the chatbot again	35 (2.7)	46 (3.6)	23 (1.8)	46 (3.6)
Talk to family/friends	52 (4.0)	37 (2.9)	17 (1.3)	42 (3.3)
Other	17 (1.3)	16 (1.2)	15 (1.2)	28 (2.2)

### Qualitative results

In total, 41 survey participants took part in follow-up interviews (medium age = 27, age range: 19-60; 63% women; 70% heterosexual; 53% Black, 23% Asian, 17% Latin American/Middle Eastern/Mixed race and 7% White other). Four main themes (Table 3) were identified: “*Chatbot as an artificial health advisor*”, “*Disclosing information to a chatbot*”, “*Ways to facilitate trust and disclosure*”, and “*Acting on self-assessment*”. Key features of Chatbot-Assisted Self-Assessment (CASA) intervention for behaviour change are presented in Table 4.

**Table 3:** Themes and subthemes on the attitudes towards chatbot-assisted self-assessment.

Theme (sub-theme)	Illustrative quotes
**Chatbot as an artificial health advisor**	
(For sensitive & embarrassing conversations)	“I feel like the first time I went there the questions were like they asked you, have you done, oral, anal...like they obviously they are asking because they need to know what the possibilities are, but I feel like those questions might be a bit embarrassing to answer face to face. So, having a chatbot might help” “If people are talking about stuff that they might feel a bit ashamed about, like specially around sexual health or just anything like that, there’s not, they’re not going to feel that kind of shame because they’re talking to a robot” “I think people would use it because you’ve got that conflict of it’s something very personal and it’s very difficult to talk to... Talking to something that hasn’t got a face might be a lot easier. So yeah, if I had an issue, I’d talk to one.”
(For anonymous advice)	“I think this an anonymous platform where no one is going to know… no one is going to know you. So, you have to take advantage of that and you know, put everything on the chat” “So, I think having something online just makes it, you know, there is that confidentiality, it’s because you’re not seeing...no one seeing your face. You are behind the screen”
(Neutral & non-judgemental)	“Take for instance, you had an appointment to take you to your doctor and you went to the hospital to see a doctor. So it’s...you are there to express yourself and explain to him better how you feel, so as he could actually go into scrutiny or, um, taking test before actual prescriptions. You know if you don’t have that, maybe you’re shy type, your timid to face him and actually explain to him in detail with the use of chatbots, you are free to express yourself from the very start to the end, so as he could understand you better and from there, I believe he will be in better position to actually make you feel comfortable” “I think is a good idea. Yeah, because again, like I come from Columbia, which is a really traditional kind of culture and is like extremely stigmatised to speak about STD’s and stuff. So, like people, never ever, ever… yeah, like if you say that you’re going to go to get tested for an STD is like, Oh my God… like, that’s crazy. But if it was like me living in Colombia or me with the mentality of somebody that lived in Colombia, I would prefer to just speak to a machine” “Obviously the chatbot isn’t going to answer all my questions, solve all my problems, but at least gives me a quick answer, some guidance”
(Convenient)	“I think you know definitely, umm, using this for the first time, was kind of bring about some level of, well I say, distrust for the first time, so telling you that you know this, you are talking about health here and want to be as accurate as possible, you know, so that we don’t get a machine telling me a negative result and all. So in order to bring about an output of accuracy, uh, I feel after imputing those details in the chatbot and it tells you the results”
(As accurate as user honest responses)	
(Personalised & relevant assistance)	“We need to be giving, you know, the most honest answer and a full response in order for it to give you an answer that is going to be accurate, so it comes from various sides I suppose.” “When it comes to more personalised risk assessment, it puts it tells you who you are, it puts you in that light of who you are. This is who I am. These are my risky behaviours and these are what, these are the things that put me at risk and with that it gives you that consciousness, that whole I need to, I need to adjust my, my risky lifestyle right? So, I think a more personal risk assessment to work better it would, it would be more effective than in general. “If they can prove to me that it’s secure, I don’t mind uhm, discussing anything, everything about my sexual health, in terms of the HIV and all that. Because I believe that personal experiences do help much more than the doctors sometimes. So, if on a chatbot you can have like a story about me, and you’ve made it so secure so that only people that are, maybe they’ve just been diagnosed, can access it.” “I’m aware that the bot is not going to make any diagnosis. I know I can’t ask it specifically questions… that do you think this is … I don’t know. So, do you think this is HIV? Do you think this is a chlamydia? Do you think… No. I know the chat bot is not going to be able to diagnose it, so I’m not expecting too much of it more than just tell me my risk level, risk exposure”
(Symptom checker, not a diagnostic tool)	“To start with, if you’re embarrassed to speak to someone about your symptoms, then a chat bot is very useful.”
(Lacking human expertise & empathy)	“Sometimes chatbots, AI, they’re just too technological, they’re just their wording is a little bit off, because it’s not like human contact.” “I don’t think I feel comfortable just telling a computer.” “I think just very sensitive issues like if anything very difficult kind of happened or something where there’s a lot of emotion around it. Like, I can’t think of an example, but maybe something where I would want to feel comfortable, like or have you know, sometimes you need a doctor to help you feel comfortable before you open up or articulate about something like stuff like that.” “I feel like people would see it that way, that there wouldn’t, there wouldn’t be any bad consequences when asking personal questions, sexual questions, STI questions. But I don’t think older generations, Catholic Ecuadorian, older generations would be able to use chatbots yet.”
(Digital inequality)	“Let me use my ethnicity couple with my area or community or residence as a case study. You know this is an era of or we call it a computer age. Ohh, we call it a digital world as the technology is advancing, we need to advance alongside the technology. I am 100% sure that you know, this is something that is actually developing on a daily basis. So within my ethnicity, I am pretty sure that if this should go. I believe everyone will embrace it because everyone is eager to use chatbots, most especially health wise.”
**Disclosing information to a chatbot**	
(Comparable to healthcare professionals)	“I don’t mind sharing my stuff with doctors anyway, so like with a sexual health chatbot, I would probably answer whatever questions, like any questions they asked me, I guess I’d answer them, because I mean definitely like when I go to the doctor, I just want an answer. So, I’m just giving all the information it needs.” “I have like a male doctor. Sometimes I wouldn’t feel comfortable telling him any sexual concerns that I have. Definitely using a chat bot in that case. I can say I have this and this. I don’t know what this is, but it’s concerning me.
(Optimal number of questions)	“I will not be precise. Anyway, I’ll give some range... I believe if some of the questions, I believe should be within the range of 10 to 15 or 15 to at most, let’s say 10 to 15, 17 is OK, because some people will be bored of the questions, you know.” “It shouldn’t be over 10 minutes. So I don’t know how many questions that would roughly be...at least about 15 to 20. I think that would be like people wouldn’t lose their what’s the word? Ideally, it would be more....to get as much of a thorough thing as possible, but if you can get the job done in 15 to 20 questions, I think that would be helpful.
(Demographic information)	“I understand that for reasons of understanding the community and better...providing a better service, that’s extremely important for the NHS, sexual health clinics, to know this data. So, I think if the chatbot was open about that with me, I wouldn’t mind at all, share my information. So, for example, if the chatbot comes to me and say before we start, we normally ask these questions because we believe that certain communities are high risk, and we want to improve our service for this communities. We’re going to ask three questions about this and that... Are you comfortable answering them? Then I would say yes, I don’t mind.” “I say it’s one that I think it’s one of those things now.... It’s like it, it seems like I don’t need to share... I feel like if it’s a reason, like, you know, I, you know, age. I feel like it makes sense, because it’s like... it’s in certain things are more important at different ages or not like, uhm, like, obviously like you start using contraception or...Uh, yeah, stuff like that.”
(Information about personal risks)	“I think it would be much more useful to have a personalised set of questions really, because I mean, that’s the whole point I guess, of the chat bot or else they would answer some questions and then you’ll have a very generalised answer.... I mean without any personal questions, it would be more difficult to understand one’s risk, one’s sexual risk practises if it weren’t a personalised.” “if I was looking for, for PrEP, for example, if I was, I wanted the chatbot to book an appointment for me, I would, I would be willing to disclose the information that the chatbot would be requiring.” They could ask more questions… the software could ask you – how do you have sex? When do you start using your condom? When is the correct time for using the condom? How should you dispose of condom?
(Information about symptoms)	“Yeah, I would not have any problem with sharing symptoms with the chatbot”. “So rather than going straight to GP, you might just put all your symptoms there and then from there they relay it to the doctor and help you book a test. And then you can go meet the doctor to see or give a bit more information or see what they possibly diagnose you with.”
(Information about minority status)	“I know a lot of gay men might have internalised homophobia, that they don’t realise, and so, that they’ll find it really hard to open up about these things. So, I think like at least an AI would bypass it because as far as they’re concerned, the information is still like they haven’t told anyone.” “I think assumptions shouldn’t be made just based on ethnicity, I think and it’s more about the behaviours one has had in their sexual history that should indicate what this question should be more... Than what’s the colour of my skin? Yeah, and I think things like probably age and again, the type of sexual relationship one has are probably a lot more informative in terms of indication of risk, than what my ethnicity is”
(Nondisclosure of personally identifying & stigmatising information)	“I don’t know if I’ll be comfortable giving like my email address or something. Maybe just because I’m paranoid and having like all these things linked to me, like all that information to my email address.” “I cannot speak for the people in general, psychologically, you know, actually say some people will be very, very unwilling to actually tell you what’s their HIV status.”
**Ways to facilitate trust and disclosure**	
Explainable AI	“Like as a customer, you don’t know what you are supposed to say, or you know, what you are supposed to do...so it would be good in the beginning if she gave you some direction, like...after she introduce herself, like, if you want to know more about symptoms... you know, give me this and that information.” “For starters, I really think it should have a kind of a menu, OK, a menu where you have a variety of things to select.”
Set the scene	“I think if the chat bot, it would just set expectations. Like for example, if the person said I like to get tested and then the chatbot comes back saying that’s fine, we’re going to ask you some questions that that you can answer if you are comfortable, if that is OK in the first, say ‘yes’” “If you see it’s just tailored to what’s a particular aspect of healthcare you might be frustrated…so, if it’s for sexual health and for let’s say contraception and all that, then you should know this is the chatbot to go to for that. Because this area is a bit broad, just STI’s, we can see a lot to explain on that. And so, it just doesn’t waste people’s time, and they feel like it’s not useful.”
Privacy & confidentiality statement	“I think I would have some reservation, I think, to use the chatbot related to sexual health, I think because it’s such a private and personal …sexual health, I mean it’s so private and personal. I definitely think I would need to have certain reassurance as I’m starting to use that feature, and I don’t know if like some level of warning or just a small line that that says how it’s processed and explaining how that’s done to kind of reassure me that it’s not going to a third party, onto a data processing agency or whatever the case may be.”
Conversationality	“If you’re going to share more information, it’s just for medical purposes. No one is going to share this information about your sexual preferences, your number of partners. I mean, it would be good to, you probably know it because you, you know, because of the data policies. But it would be good to like … for the bot to reinforce that, to reassure that for you, it’s just for medical matters.” “You could definitely develop the beginning cause Pat [chatbot] did leap into something very quickly. I was like, alright, calm down! It went from this question to the response, here’s your response, your answer, and I was like. I think you could definitely build in that little bit more around…. You can be like it says ‘Hi, I’m Pat. I’m still learning blah’. But then it can be like, ‘let’s talk about you’ and you can answer a few questions... You maybe just think, oh, we could have a bit of chat you know, a little bit more chat.”
Automated language translation	“I think maybe at the end of the conversation to have like a nice goodbye or something versus just like here is a link to this, because I think it’s a lot more fun when it has something like that.” “Well, for my ethnic group, I would probably provide an option to switch to a different language, you know, I would consider that because all my friends speak English quite fluently, but I hear from friends, that other people do not always speak English very fluently.” “I think the chatbot might be a barrier for different ethnicity populations if only English version is provided. If other language version is provided in their own language, mother language or instant translation is provided for them, I think it would encourage the minorities to use the service. Sometimes the software is not well developed…and minority people might find this is not… for Chinese population, the website, the application might have many pages with Chinese but if you go into the website, some of the services might only be provided in English…this may prevent people from further usage.”
Pleasure & enjoyment	“I would use it to get more information. Umm. Yes, things like that, how to, I don’t want to be, um, too intimate or explicit with some of my words, but the use of condom? I would think I would ask the chatbot.... How do I enjoy sex using condom?” “I think it would be good if the chat bot could like, had affiliations with other places that produced more specific content or whatever, like I don’t know, sometimes there’s like online videos or there could be a web series about like, I don’t know, like how to pleasure yourself, how to pleasure, a vagina like, stuff like that.”
Normalising & destigmatising language	“Like kind of normalising the conversation around sexual... like...trying to just get people to use it like any other health service, like the way you just go to the doctor if you’re not feeling well. Something that you should not be like trying to hide or anything. Yeah. I mean, I’m just, you know, ask the chat bot for some advice’. “Neutralising it and normalising it, it could let you know, oh this has been asked by so many people so do not worry or this is normal… that it could give you facts instead of saying this is wrong or this is right - it could just give you facts - this is what humans do. There’s no right or wrong... say something like ‘oh do not worry, this question is asked often’ or ‘do not worry this is not something you have to worry about, it’s normal’. That’s the type of reassurance, reassurance I would be looking for. If I need to look for the answers to such a tricky question to such tricky topics, HIV or even like chlamydia or gonorrhoea, things like that. I feel like if the chatbot is able to talk about it like it has happened to many people, I wouldn’t feel so bad about my own case.”
Responsiveness & sympathy	“It’s hard for AI to feel, to emulate human emotions. But I feel like reassurances. Something easy to copy or something like ‘oh I’m here for you. I understand. I understand what you’re going through’ or even by giving us facts like ‘do not worry, this has happened too many’, something like that.” “Because you are probably scared... I mean if you are going to talk to a bot, you are scared, you’re concerned about something, so yes, you need to… I mean, you’re worried about, you need to feel like…. more personal, more ‘warm’. So yeah, I think empathy is important.”
Behavioural instructions & signposting	“So, like having, like, instant information. Umm, what else? Again, as I said to you, like if I need to have some other type of different treatment that you could help me where I could get the treatment, how long the treatment takes... like there just needs to be more accessibility of information and of knowledge of how to do the STD testing. Even I think there is an option of you getting tested at home.” “I think it would be good if the chatbot had links or affiliations to any charities that may support black, queer people like stuff, like that and stuff...if they advised me to talk to a professional like it would be good if then it could say, you know, seek out this charity or this resource.”
**Acting on self-assessment**	
Disengagement & denial	“They might receive from the chatbot that they have a low percentage of transmitting the STI and they may stop using professional services.” “And if I was scared, being stigmatised …’cause I feel like that that comes with it. So maybe I guess what I’m trying to communicate is that there is also that denial part of things. So, like if I see that, then I’ll go into denial and be like I’m not gonna test. I’m just gonna live my life. I’m not sure. I guess that depends on the personality of the person more than on the culture, I think.”
Seeking confirmation from health professionals	“If I have 60% of risk of transmission of STI I might be seeking professional medical help, after using the chatbot service. And sometimes people might be afraid of transmissions but if they know that they have used a condom correctly, the prescription has been given correctly, they might receive from the chatbot that they have a low percentage of transmitting the STI and they may stop using professional services and this will take pressure from the NHS.” “If it was me personally, and it told me something like that, I might want to talk to a doctor afterwards. But like, I think cause sometimes there might be a bit more… nuanced than what it covers, but I think it’ll give you the general like I guess what the kind of sex, and how many partners you’ve had or something? It’s I guess a bit of a formula, but you might in talking to a doctor uncover some other stuff that might mean you could be more at risk or whatever.”
Booking appointment & accessing services	“I think the chatbot for me, the ideal chatbot is where a chatbot that would book appointments for me, that would actually put me in touch with their service, rather than just telling me how to access the service. A more active, proactive chatbot rather than a passive chatbot. And therefore, this chatbot would need really to raise, assess risks. They would, they would, assess risks, and give me recommendations, but in the end they would all.... I don’t know, they would just book appointment for me and judging if I was at risk or not. So, I guess my answer is yes, it would be beneficial if in the end the chatbot actually linked me with sexual health clinic directly.” “I would much prefer is having if I’m having that chat bot conversation, I would also want to be able to book that appointment, test, whatever the case might be directly with within the environment of the chat bot.”
Order home self-testing kit	“So you could actually put a bit of linkage to where they could get the HIV self-testing kits because for some people they will want to call and maintain a level of confidentiality to an extent too. You could instead of just saying you could go get tested, you could also tell them what you can do right? What you could tell them how they could access more self-care services.”

**Table 4:** Key features of Chatbot-Assisted Self-Assessment (CASA) intervention for behaviour change.

Key features of CASA	Brief description of the feature
**Introduction**	The chatbot should offer a concise introduction that clearly explains its purpose, the scope of the assessment, and any limitations users should be aware of. This sets the stage for user engagement and informed participation.
**Privacy & confidentiality**	The chatbot must transparently inform users about the data collection process, including what personal data is gathered, how it is stored, and with whom it may be shared. This ensures that users feel secure and confident in the privacy of their information.
**Conversationality**	The chatbot should engage users with follow-up questions that are relevant and personalised to their health assessment. This interactive approach can help maintain user engagement and provide more accurate assessments.
**Language translation**	The chatbot should be equipped to operate in multiple languages, ensuring accessibility for users from diverse linguistic backgrounds. This broadens the reach of the assessment tool.
**Destigmatising language**	The chatbot should employ positive and normalising language when asking about behaviours or conditions that might be sensitive. This approach reduces the potential for stigma and encourages honest responses.
**Sympathetic language**	The chatbot should acknowledge the emotional difficulty of discussing sensitive topics. By responding with empathy, the chatbot can create a supportive environment that fosters trust and openness.
**Question explainability**	For each question asked, the chatbot should provide users with an explanation of its relevance to the overall health assessment. This transparency helps users understand the purpose behind each query, enhancing their engagement.
**Number of questions**	The assessment should be concise, typically consisting of 15 to 20 questions. This balance ensures comprehensive coverage without overwhelming the user.
**Signposting**	After the assessment, the chatbot should offer clear behavioural guidance based on the user’s results. These tailored recommendations support users in taking appropriate next steps for their health leading to behaviour change.

#### Chatbot as an artificial health advisor.

Overall, the participants had a positive view of health chatbots. They perceived them as a convenient and quick source of information for their medical questions, as well as an engaging and interactive tool that could improve their understanding of illnesses, infections, health risks, and healthcare services. The participants agreed that chatbots could raise awareness about various health issues and improve knowledge about available clinical interventions and services. However, some participants were concerned that chatbots offered limited content, and were not comprehensive sources of information on all health-related topics. There was a divide in opinions regarding the ideal number of chatbots, with some participants preferring a single chatbot that covered a wide range of sexual and reproductive health topics, while others believed that multiple, topic-specific chatbots would be more accurate and precise. The latter group felt that a specialised chatbot for each topic, such as STI screening, vaccinations, or contraception, would provide more in-depth information.

The capabilities of health chatbots were compared to those of healthcare professionals. Limitations, such as the robotic, unnatural feel of chatbots were acknowledged. Emphasis was placed on the need for sensitive, empathic and compassionate language despite being a computer program. The framing of questions and response options was deemed crucial for inclusiveness and avoiding bias. Specifically, chatbots require plain language accessible to all users, including non-native English speakers, unlike healthcare professionals, who can tailor language to each patient. Participants were interested in using chatbots for sensitive topics such as STI symptoms and other taboo issues. Some saw chatbots’ lack of cultural awareness as a limitation, while others saw it as a fair aspect for all users.

Participants viewed chatbots as a suitable option for private conversations about sexual and reproductive health, which they considered difficult to have face-to-face with a healthcare provider. The advice from chatbots was perceived as non-judgmental and impartial due to their lack of human preconceptions. Chatbots were believed to be best used for anonymous interactions and personalised advice, where users could ask intimate questions and receive a response or recommendation for specific actions. Some compared chatbots to virtual ‘risk calculators’ or personal risk assessment tools in the form of online questionnaires or health surveys. Chatbots that asked a series of questions imitating a patient-doctor conversation were considered more personally relevant than those that only provided information. One participant framed the interaction with the chatbot as a “*let’s talk about me*” type of conversation where health-related personal concerns could be explored. Although chatbots were not seen as capable of diagnosing diseases, they were viewed as useful as a symptom checker or for assessing eligibility for preventive measures such as screening or vaccination. The majority believed that chatbots that asked personal questions for self-assessment were as accurate as the users’ responses to those questions. Thus, participants emphasised the need for chatbots to explain why each question is being asked to understand the relationship between the chatbot questions and their health status.

The main obstacles to the use of chatbots in healthcare were their comparison to human healthcare professionals. Although they were deemed suitable for sensitive and embarrassing topics, many participants felt that chatbots could not provide the reassurance and expertise necessary for a reliable and full consultation. As a result, participants viewed chatbots as most useful for health education and promoting adequate healthcare services, allowing users to make informed decisions about accessing healthcare. There were also concerns that people were unfamiliar with the technology and therefore unwilling to use it, particularly older adults or ethnic minorities who were more accustomed to in-person interactions. A general agreement was reached that most people lacked knowledge of how chatbots work and who, if anyone, was behind them.

#### Disclosing information to a chatbot.

Participants believed that the ideal number of questions asked by a chatbot should be between 10 and 20. Chatbots that asked only a few questions were viewed as too generic and less relevant to the user, resulting in inaccurate recommendations. If the chatbot involved too many questions, participants feared it would lead to drop-out and non-disclosure. If the chatbot was anonymous, participants were more willing to share personal health information, often feeling just as comfortable answering questions as they would with a healthcare professional. Some participants also felt more at ease discussing sensitive sexual and reproductive health topics with a chatbot compared to a human healthcare professional, particularly if they felt uncomfortable with the professional’s gender or religious background.

Participants expressed mixed feelings about revealing personally identifiable information to chatbots. While they were generally willing to discuss sensitive health topics anonymously, many felt uneasy about sharing personal details like their name or phone number. However, they felt that chatbots that asked personalised questions about health risks, such as the number of sexual partners or condom use, were more relevant and provided more accurate recommendations. Disclosing demographic information like age or sex was generally acceptable, but there was concern that questions about minority status could lead to distrust if not properly explained. Most participants agreed that chatbots should focus on behaviour related to health risks rather than demographic information to avoid further marginalization. Participants were comfortable discussing symptoms with chatbots, but acknowledged that users may not fully understand their symptoms. Finally, participants felt that sensitive topics requiring high levels of empathy, such as sexual abuse or sexual problems, were not suitable for discussion with a chatbot.

#### Ways to facilitate trust and disclosure.

The participants stressed the importance of certain design features in chatbots to improve their trust in the intervention and encourage more truthful disclosure of health information. They suggested that chatbots should present information about their creators, how they work, and the reliability and accuracy of their information, based on research or evaluation. A clear introduction or information page was seen as favourable, explaining the chatbot’s purpose, how it engages in conversation, what aspects of health it is designed for, and its limitations, such as the inability to diagnose diseases. The participants believed that setting clear expectations for chatbot usage would increase their confidence in the technology. This introduction should also include information about confidentiality, how user data is used and stored, and who has access to it.

Participants emphasised the importance of chatbots’ conversational ability and speed of response as key factors that set them apart from other health interventions such as static websites. They believed that a conversational approach makes the experience more engaging, and rapid responses were seen as more satisfying. However, a high frequency of messages from the chatbot was seen as overwhelming and not helpful for users with lower health literacy. Participants suggested that chatbots should have a polite and respectful personality, and should be able to initiate and end conversations in a manner that mirrors human interaction, using expressions like ‘hello’, ‘thanks’, ‘my next question is’, and ‘goodbye’.

The importance of using neutral and medically accurate language in the chatbot design was emphasised throughout the interviews. The chatbot should be appealing to individuals who may be uncomfortable talking to healthcare professionals by using positive, destigmatising, and normalising language when discussing behavioural health risks, such as sexual activity or regular health check-ups. The chatbot should show empathy towards users, offering contextual prompts for potentially difficult or embarrassing questions giving users the option to skip questions. Rather than mentioning the personal risk of disease, it was recommended the chatbot present information about “*eligibility*” for screenings, vaccinations, or doctor consultations. Participants strongly discouraged that chatbots inform users about disease risks. Additionally, to create a positive experience, chatbots should recognise the pleasure and enjoyment in various activities related to health and well-being rather than only discussing health risks. Chatbots should provide clear recommendations for action based on self-assessments, informing users what steps to take next, when to seek healthcare, and how to access it. Many participants preferred the chatbot to have an appointment booking feature if it recommends a professional consultation or health screening.

#### Acting on self-assessment.

There were mixed opinions and reactions about the chatbot’s recommendations for further action. Some were worried that users might disregard or reject the need for healthcare services, despite the screening recommendations, due to cultural and personal factors, and stigma perceptions. However, most participants believed that the chatbot could be a starting point for accessing healthcare services. Many said they would seek clarification from healthcare professionals if the chatbot recommended tests or treatments. Some participants felt that having a conversation with the chatbot, discussing personal and medical questions, could help prepare users for better consultations with healthcare professionals by promoting familiarity with potential questions. Some believed the suggestions from health chatbots would motivate them to schedule sexual health check-ups or utilise at-home STI screening options. Many felt they would respond positively to the chatbot’s suggestions but would also verify their consistency by re-engaging with the chatbot. There was a concern that the chatbot’s recommendations should not create any additional barriers for individuals already seeking screening or vaccinations. Thus, the suggestions should be presented positively and encouragingly.

## Discussion

This study is the first to investigate the design features of a health chatbot for marginalised populations, specifically racialised minorities. Results show moderate acceptability and neutral-to-positive attitudes toward health chatbots, and a willingness to share demographic and health information as part of self-assessment. There was no significant difference in comfort levels among ethnic groups, indicating the potential usefulness of this intervention format in underserved populations. However, participants were less comfortable disclosing personally identifiable information, highlighting the importance of anonymity in chatbot designs. Previous chatbot experience, overall acceptability, and positive attitudes were the most significant predictors of information disclosure, suggesting that hesitancy to interact with chatbots could be due to the lack of familiarity, previous adverse experiences or negative perceptions of this technology. Design principles for chatbots in include privacy, transparency, conversationality, and multilingual translation for overall acceptability and engagement in diverse populations.

Our findings suggest that people’s attitudes, beliefs, and perceptions are more important than demographic factors such as gender, age, or ethnicity in determining their comfort level with disclosing health-related information to chatbots. This contradicts previous findings that younger people are more accepting of chatbots [29]. However, the COVID-19 pandemic may have had an impact on people’s attitudes towards digital health services, particularly conversational channels such as webchats [30]. Additionally, since this study focused on sexual health matters, the participants may have had previous exposure to digital interventions, representing a generally younger and more technologically literate population, which could explain relatively higher levels of chatbot awareness and previous use in our sample as compared with health conditions that affect older populations. Our studies support the findings that AI is initially viewed with caution and reservations, particularly by those with a limited understanding of health chatbots [31,32]. This aligns with the Diffusion of Innovation theory, which states that the spread of new technologies varies through cultures and depends on perceptions of relative advantages, compatibility, complexity, trialability, and observability [33]. Thus, moderate acceptability and neutral attitudes to chatbots are expected, especially amongst those with no previous experience with them.

### Design principles for chatbot-assisted self-assessment

To promote positive attitudes and increase engagement with health chatbots among ethnic minorities, it is essential to clearly state their purpose and capabilities in accessible language. Chatbots must address specific problems and individual concerns related to trust and confidence before engagement is initiated. We found that transparency in how chatbots work and who is behind them is crucial for building trust, which is supported by other studies on AI [34,35]. Attitudes towards health chatbots may be influenced by concerns of bias or discrimination towards certain ethnic groups. Our study found that participants emphasised the importance of training AI algorithms using diverse and unbiased data. This is especially important for people from ethnic minority backgrounds who may need clear assurances that health chatbots are non-discriminatory. Positive experiences with health chatbots can foster trust among individuals who fear discrimination from traditional healthcare providers and subsequently increase their engagement with healthcare services. Therefore, developing an evidence base and guidelines for the ethical use of health chatbots for various minoritised communities is likely to increase trust, influence the initial experience with chatbots and promote positive attitudes. We emphasise that the co-production of Al-led health interventions, such as chatbots, involving diverse communities in terms of age, gender identity, ethnicity, socioeconomic status, religion and disability, needs to become a standard practice to eliminate any potential bias and optimise overall acceptability and engagement [36–38].

Participants in our study expressed concerns about the confidentiality of their data when using health chatbots. These concerns are well-documented and are likely to affect trust in this technology [39]. An explicit reassurance about data security could improve users’ willingness to disclose health-related information. Participants also expressed concerns about the disclosing demographic information, especially when it comes to ethnicity, as it could reinforce negative stereotypes. Previous research has highlighted that individuals who identify or have characteristics dissimilar to the majority population may experience marginalisation and a perceived social distance, which may affect their willingness to disclose information [40]. Thus, health chatbots should explain why they are requesting such information; users will be more likely to disclose this information if it leads to more tailored and specific information. Both anonymity and confidentiality must be considered, particularly for stigmatised conditions such as HIV or drug use, where users may be hesitant to provide personally identifiable information. These concerns may be higher in those from ethnic minorities who are more likely to distrust healthcare professionals [41]. Due to the risk that poorly designed chatbots may introduce an additional barrier to healthcare if users experience an adverse reaction, chatbot designers need to consider all aspects of marginalisation and intersectionality when sensitive questions are being asked.

Our research found a strong preference for the conversationality of health chatbots which could be understood as the ability to hold a natural-sounding conversation. This could help to create a more engaging and personalised experience for users and to build trust and rapport, making them more likely to engage with the chatbot and disclose personal information. Conversational chatbots can help to improve the user experience by providing more relevant and tailored information and support. There is a potential that large language models, such as ChatGPT, could improve the conversationality of chatbots while answering personalised questions about health and well-being [42]. However, more research is needed to evaluate these emerging technologies and ensure that they are ethically designed and implemented.

### Strengths and limitations

We employed diverse recruitment methods to include underrepresented communities in AI research, which were represented in our sample. Our mixed-methods approach facilitated data triangulation and provided a more comprehensive understanding of issues related to CASA interventions [43]. Participants were asked to interact with a sexual health chatbot during interviews to ensure that opinions were based on experience rather than hypothetical scenarios. However, there were limitations to the study. Ethnic minorities are a heterogeneous and diverse population with a wide range of views on health chatbots due to cultural, linguistic, and other aspects of marginalisation. Thus, there were diverse views of both sexual health and AI. People of various cultures may formulate distinct and unique views on health chatbots, which may also differ according to the type of disease or condition as well as their perceptions of severity and stigma [44]. Future research should further explore attitudes among individual ethnic groups to identify those differences and explore the willingness to disclose health-related information to chatbots across several health conditions. In addition, individuals with limited access to technology and healthcare services may have been under-represented in our sample, despite our use of diverse recruitment methods. While we collected data on educational attainment, self-reported chronic conditions, and language spoken at home as potential indicators of marginalisation, future research should also consider measuring socioeconomic status and access to technology, such as smartphone ownership, to better understand those who may be most vulnerable to poor health outcomes. Our study highlights the need to establish methods for AI chatbots to serve those from lower socioeconomic backgrounds. In addition, the data were collected before the widespread advancements in large language models, and it is possible that since then, more people have become familiar with chatbots, such as ChatGPT, and their attitudes towards the use of this technology in health are different. As such, future research should monitor acceptability and hesitancy towards conversational AI in ethnic minorities to predict their engagement and uptake.

This research shows that CASA intervention is acceptable in ethnic minorities and can be used to engage in private conversations about sensitive and health-related issues. It highlights a strong preference for education around available services, such as screening or vaccination, based on self-assessment, rather than estimation of disease risk. People from ethnic minorities may be less likely to accept AI-led ‘risk calculators’ or risk prediction tools, and participants in our sample reacted differently to the chatbot risk appraisal of STIs, indicating that presenting ‘risk information’ may not lead to the desired action. To be effective, CASA interventions should provide simple but informative explanations of the role and function of health chatbots, their confidentiality and privacy, and accurate multilingual translations. Previous research demonstrated that ‘sensitivity signalling’ had a positive impact on user disclosure of personal information to chatbots [45]. Thus, to promote transparency and trust, chatbots for self-assessment should justify each health-related question and engage in ‘positive conversations’, offering encouraging and empowering responses and linking users with organisations that provide further support. However, more research is needed to understand the impact of health chatbots, using the CASA approach, on individual behaviours and healthcare services for each minoritised community to systematically evaluate this type of intervention. Guidelines for patient-facing AI-led health chatbots should consider using behaviour change theory within their designs, as outlined for other AI systems [46], so that the mechanisms of action for chatbots are sufficiently understood. Our study also highlights the importance of addressing concerns about bias and discrimination in developing health chatbots for ethnic minorities, as positive experiences with chatbots can foster trust and increase engagement with healthcare services.

In conclusion, our study offers evidence for a conversational intervention in which users assess their health via relevant questions posted by a chatbot. CASA interventions could reduce stigma and break the taboo around topics that require privacy. They can also allow users to prepare for medical consultations by explaining questions that healthcare professionals frequently ask. However, this type of intervention is unlikely to benefit people with poor access to technology or the internet. Therefore, we emphasise the need for outreach services to reach disadvantaged groups, ‘seldom heard’ individuals and those with unmet needs. Such services could use multilingual chatbots to reduce language barriers and increase awareness of healthcare services. Although more evidence is needed, we anticipate that a conversational intervention carefully co-designed with members of ethnic minorities may reduce some barriers to healthcare utilisation that are typically associated with how people perceive their health and whether they are aware of services that are available to them.

## Supporting information

S1 File Survey questionnaire. (DOCX)

S1 Table Means and standard deviations for comfort disclosing sensitive, demographic and personally identifiable information to chatbots. (DOCX)

S2 Table Means and standard deviations for comfort disclosing information to chatbots by ethnicity. (DOCX)
