# Supplementary material for: Chatbot -assisted self-assessment (CASA): Co-designing an AI -powered behaviour change intervention for ethnic minorities
Source: PLOS Digit Health. 2025 Feb 13;4(2):e0000724. doi: 10.1371/journal.pdig.0000724 (PMC11824973; doi:10.1371/journal.pdig.0000724)
Supplement: S1 File — (DOCX) [file pdig.0000724.s001.docx]

**Supplementary file**

**Survey questionnaire**

Before we explore your views about online platforms for sexual health advice, we would like to ask you a few questions about yourself. All the answers you provide are confidential.

**What year were you born?** _____________________________________________________

- Prefer not to answer

**Do you currently live in the UK?**

o Yes

o No, please specify where you live: _______________________________________________

- Prefer not to answer

**What is the main language you speak at home?**

o English

o Other, please specify: ________________________________________________

- prefer not to answer

**How do you identify?**

o Man

o Woman

o Non-binary or gender diverse

o Other, please specify: _________________________________________

- prefer not to say

**What sex were you assigned at birth?**

o Male

o Female

o Undetermined

**Do you have any physical or mental health condition or illness that has lasted for 12 months or more?**

o Yes

o No

o Prefer not to say

**What is your highest educational qualification?**

o No formal qualifications

o One to four GCSEs (any grade), O ’levels or equivalent

o Five or more GCSEs (grade A* to C) or equivalent (including intermediate apprenticeships)

o Two or more A levels, or equivalent

o Higher education below university degree

o Undergraduate degree or equivalent

o Postgraduate degree or equivalent

o Other qualification, please specify: _______

**What is your ethnic group?**

o Asian: Indian

o Asian: Pakistani

o Asian: Bangladeshi

o Asian: Chinese

o Asian: Any other Asian

o Black: African

o Black: Caribbean

o Black: Any other Black/African/Caribbean background

o White: English/Welsh/Scottish/Northern Irish/British

o White: Irish

o White: Roma or Irish traveller

o White: Any other White background

o Mixed: White and Black Caribbean

o Mixed: White and Black African

o Mixed: Any other Mixed/ Multiple ethnic background

o Other: Arab

o Other: Latin American

o Any other ethnic group, please describe: ______

**Questions about chatbots**


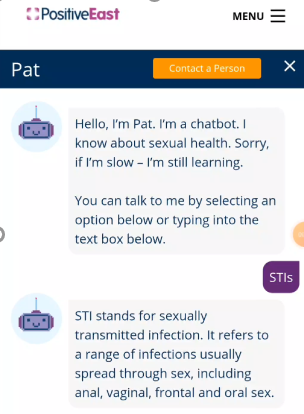


**WHAT IS A CHATBOT?**

A chatbot or ‘bot’ is **a computer program that simulates natural human conversations with users**. Users communicate with a chatbot via an online interface or by voice, like how they would talk to a real person. Chatbots interpret and process user's words or phrases and give an instant pre-set answer. Sometimes chatbots are used to respond to questions about health and wellbeing. For example, you can ask a chatbot “What are the symptoms of common flu?” and it may respond “The symptoms of common flu are fever, cough and muscle ache.” On the right is an example of a chatbot called Pat, from the Positive East charity that is used for sexual health advice.

Now, we would like to ask you a few questions about chatbots.

**Have you heard of a bot or chatbot before this survey?**

o Yes

o No

o Not sure

**Have you ever used a bot or chatbot before?**

o Yes

o No

o Not sure

**Have you ever used a bot or chatbot for any information about your health or healthcare services?**

o Yes

o No

o Not sure

**If a chatbot for health advice was available to you today, how likely would you be to use it?**

o Very unlikely

o Unlikely

o Not sure

o Likely

o Very likely

**Your views on health chatbots**

Next, we would like to ask about your views on health chatbots.

**Please indicate to what extent you agree or disagree with the following statements:**

|  | **Strongly agree** | **Agree** | **Neutral** | **Disagree** | **Strongly disagree** |
| --- | --- | --- | --- | --- | --- |
| A health chatbot could be very convenient. | o | o | o | o | o |
| I am confident that I would be able to use a health chatbot. | o | o | o | o | o |
| I would use a health chatbot for minor things such as booking medical appointments. | o | o | o | o | o |
| I would use a health chatbot if it was endorsed by the NHS. | o | o | o | o | o |
| I would use a health chatbot if my doctor recommended it. | o | o | o | o | o |
| A health chatbot could answer my questions when I cannot see a doctor. | o | o | o | o | o |
| I would trust the information provided by a chatbot. | o | o | o | o | o |
| Talking to a health chatbot would be frustrating. | o | o | o | o | o |
| I worry that a health chatbot would not understand my questions. | o | o | o | o | o |
| I would use a chatbot when I am embarrassed to talk to a doctor. | o | o | o | o | o |
| I would use a chatbot to learn about my health, symptoms and clinical tests. | o | o | o | o | o |

**Sexual health questions**

The following questions are about your sexual health. Remember, all the answers you provide are completely anonymous.

I worry that a health chatbot would not understand my questions.

In the past 12 months, how many times have you searched the Internet for any information related to your sexual health? For example, searches might be for information on sexually transmitted infections, HIV and health services.

___________

How comfortable are you to discuss your sexual behaviours with a doctor or any other health professional?

o Very comfortable

o Somewhat comfortable

o Neither comfortable nor uncomfortable

o Somewhat uncomfortable

o Very uncomfortable

In comparison to people of your age, how would you rate your chances of getting a sexually transmitted infection?

o Much below average

o Below average

o Average

o Above average

o Much above average

Who do you usually have sex with?

o Men

o Women

o Both men and women

o Non-binary or gender diverse people

o Prefer not to say

o Other, please specify: ____

In the past 12 months, how many people have you had sexual activity with? Please insert a number: ____________

- Prefer not to answer

**Sharing information with a chatbot**

Some chatbots are designed to help you make decisions about your health, like whether you need to test for sexually transmitted infections. To do this, they need to understand the type of questions you may ask, and the kind of information you are willing to share with a chatbot.

**If a free chatbot for sexual health advice was available to you today, what kind of question might you ask? [Please write down as many as you can think of in the box below]**

Some chatbots may be able to calculate your risk of getting a sexually transmitted infection (STI). To do so, they need to ask about personal information to understand your potential exposure. This next question explores what personal information you would be willing to share with a chatbot.

**How comfortable would you be to share personal information with a chatbot in order to access sexual health services regarding the following topics?**

|  | **Very comfortable** | **Somehow comfortable** | **Neither comfortable nor uncomfortable** | **Somehow uncomfortable** | **Very uncomfortable** |
| --- | --- | --- | --- | --- | --- |
| About having symptoms of a sexually transmitted infection (STI) | o | o | o | o | o |
| About being diagnosed with an STI in the past | o | o | o | o | o |
| About the date of your last STI screening | o | o | o | o | o |
| About being in contact with anyone diagnosed with an STI | o | o | o | o | o |
| About the date of your last sexual intercourse | o | o | o | o | o |
| About the number of sex partners since your last STI screening | o | o | o | o | o |
| About your use of dating apps | o | o | o | o | o |
| About your use of condoms | o | o | o | o | o |
| About receiving money or goods in exchange for sex | o | o | o | o | o |
| About your use of illicit /recreational drugs | o | o | o | o | o |
| About your level of worry about having an STI | o | o | o | o | o |
| About being diagnosed with a mental health condition | o | o | o | o | o |
| About your age | o | o | o | o | o |
| About your gender assigned at birth | o | o | o | o | o |
| About your ethnicity | o | o | o | o | o |
| About the ethnicity of your sexual partners |  |  |  |  |  |
| About your sexual orientation | o | o | o | o | o |
| About your gender identity | o | o | o | o | o |
| About your name | o | o | o | o | o |
| About your email address | o | o | o | o | o |
| About your telephone number |  |  |  |  |  |

**Responding to chatbot advice**

Some chatbots may provide recommendations for sexual health screening, such as an HIV test. The following questions asks you about your response to four different chatbot’s recommendations.

**Please let us know how you would react to the following message from a chatbot.**

**Imagine a chatbot told you: “Based on your answers, you are at lower risk of sexually transmitted infections compared to people of your age”. What would be your reaction?**

o Visit a sexual health clinic or see your GP

o Call the sexual health clinic via telephone to seek more information

o Order a test kit online that can be delivered home

o Do nothing

o Talk to the chatbot again

o Talk to a friend or family member

o Other reaction, please specify: ____________________________________________

**Imagine a chatbot told you: “Based on your answers, you are at average risk of sexually transmitted infections compared to people of your age”. What would be your reaction?**

o Visit a sexual health clinic or see your GP

o Call the sexual health clinic via telephone to seek more information

o Order an online test that can be delivered home

o Do nothing

o Talk to the chatbot again

o Talk to a friend or family member

o Other reaction, please specify: ____________________________________________

**Imagine a chatbot told you: “Based on your answers, you are at higher risk of sexually transmitted infections compared to people of your age”. What would be your reaction?**

o Visit a sexual health clinic or see your GP

o Call the sexual health clinic via telephone to seek more information

o Order an online test that can be delivered home

o Do nothing

o Talk to the chatbot again

o Talk to a friend or family member

o Other reaction, please specify: ____________________________________________

**Imagine a chatbot told you: “Based on your answers, you might be at risk of sexually transmitted infections, but it is too early to test” What would be your reaction? [Note: it is unlikely for an STI test to show positive results of infection if the test was conducted shortly after sexual intercourse.]**

o Visit a sexual health clinic or see your GP

o Call the sexual health clinic via telephone to seek more information

o Order an online test that can be delivered home

o Do nothing

o Talk to the chatbot again

o Talk to a friend or family member

o Other reaction, please specify: ____________________________________________

**Your views on sexual health chatbots**

Now, we would like to ask about your views on sexual health chatbots and sexually transmitted infections (STIs)/human immunodeficiency virus (HIV).

**Please indicate to what extent do you agree or disagree with the following statements:**

|  | **Strongly agree** | **Agree** | **Neutral** | **Disagree** | **Strongly disagree** |
| --- | --- | --- | --- | --- | --- |
| A sexual health chatbot could help me to find information about STIs and HIV. | o | o | o | o | o |
| I would use a chatbot that could estimate my risk of STIs and HIV. | o | o | o | o | o |
| I would not trust any information provided by a chatbot about my sexual health. | o | o | o | o | o |
| I would test for STIs and HIV if a chatbot suggested it. | o | o | o | o | o |
| I will use a sexual health chatbot if my doctor recommends it. | o | o | o | o | o |
| A sexual health chatbot would help me to decide if I need to test for STIs and HIV. | o | o | o | o | o |
| I would feel embarrassed talking to a chatbot about my sexual health. | o | o | o | o | o |
| I would be worried about confidentiality while discussing my sexual health with a chatbot. | o | o | o | o | o |
| I would not share any information with a sexual health chatbot because I am concerned about my privacy. | o | o | o | o | o |
| I think a sexual health chatbot would give accurate information. | o | o | o | o | o |
| I would disclose personal information to a chatbot if it helps me to understand my risk of STIs and HIV. | o | o | o | o | o |
| A sexual health chatbot would increase my anxiety about having an STI. | o | o | o | o | o |
| A chatbot could prepare me for an uncomfortable conversation with a doctor about my sexual health. | o | o | o | o | o |

**If a chatbot for sexual health advice was available to you today, how likely would you be to use it?**

o Very unlikely

o Unlikely

o Not sure

o Likely

o Very likely
