# Supplementary material for: Chatbot -assisted self-assessment (CASA): Co-designing an AI -powered behaviour change intervention for ethnic minorities
Source: PLOS Digit Health. 2025 Feb 13;4(2):e0000724. doi: 10.1371/journal.pdig.0000724 (PMC11824973; doi:10.1371/journal.pdig.0000724)
Supplement: S1 Table — (DOCX) [file pdig.0000724.s002.docx]

**S1 Table. Means and standard deviations for comfort disclosing sensitive, demographic and personally identifiable information to chatbots**

| **Variable** [*5-point Likert scale*] | **Comfort disclosing sensitive information [mean; SD]** | **Comfort disclosing demographic information**  **[mean; SD]** | **Comfort disclosing personally identifiable information**  **[mean; SD]** |
| --- | --- | --- | --- |
| Gender     Male     Female     Non-binary/other  Gender identity different than at birth     Different gender identity     Same gender identity  Ethnicity     White British     White Irish     White Roma or Irish Traveller     White Other     Mixed White and Black Caribbean     Mixed White and Black African     Mixed Other     Asian Indian     Asian Pakistani     Asian Bangladeshi     Asian Chinese     Asian Other     Black African     Black Caribbean     Black Other     Arab/Middle Eastern     Latin American     Any Other  Main language spoken at home     English     Other  Education     No formal qualifications     5 or less GCSE/O level or equivalent     2 or more A levels or equivalent     Higher education below degree level     Degree or equivalent     Postgraduate degree or equivalent  Other  Mental or physical health condition     Yes     No  Prefer not to say   Ever heard of a chatbot     Yes     No/Not sure  Ever used a chatbot     Yes     No/Not sure  Ever used a chatbot for healthcare     Yes     No/Not sure  Likelihood of using chatbot for healthcare     Unlikely     Not sure     Likely  Preference of sexual partners     Opposite gender     Same-sex male     Same-sex female     Both genders     Non-binary/diverse  Number of sexual partners in last 12 months     Zero     One     Two or more  Self-rated risk of STI compared to others     Below average     Average     Above average  Comfort discussing sexual health with HCP     Comfortable     Neither comfortable nor uncomfortable     Uncomfortable | 2.23 (0.94)  2.28 (0.97)  1.92 (0.93)  1.84 (0.57)  2.27 (0.96)  2.16 (0.94)  2.65 (1.42)  2.56 (1.22)  2.25 (1.01)  1.81 (0.54)’  2.34 (0.67)  2.25 (1.00)  2.21 (0.95)  2.67 (0.93)’  2.25 (1.07)  2.23 (0.91)  2.25 (0.84)  2.20 (0.93)  2.30 (0.96)  2.35 (1.21)  2.53 (0.96)  2.45 (1.07)  2.45 (1.07)  2.24 (0.95)  2.28 (0.98)  3.13 (1.13)’  2.56 (1.08)  2.17 (0.99)  2.39 (0.99)  2.24 (0.92)  2.16 (0.98)’  2.40 (1.03)  2.29 (1.08)  2.23 (0.92)  2.77 (0.96)  2.16 (0.92)  2.59 (1.02)  2.13 (0.91)  2.56 (1.01)  2.05 (0.81)  2.33 (0.99)  2.67 (1.19)  2.41 (0.88)  2.03 (0.82)  2.22 (0.93)  2.38 (1.21)  2.28 (1.09)  2.12 (0.83)  2.05 (0.93)  2.55 (1.06)  2.14 (0.93)  2.13 (0.85)  2.28 (0.98)  2.23 (0.89)  2.14 (0.95)  2.09 (0.90)  2.68 (0.87)  2.44 (1.04) | 1.72 (0.91)  1.60 (0.87)  2.01 (0.76)  1.82 (0.65)  1.65 (0.89)  1.62 (0.95)  2.51 (1.63)’  2.13 (0.98)  1.58 (0.94)  1.50 (0.64)’  1.91 (0.60)  1.73 (0.96)  1.60 (0.87)  1.93 (0.90)  1.77 (0.93)  1.72 (0.87)  1.92 (0.87)  1.55 (0.78)  1.61 (0.83)  1.63 (0.80)  1.46 (0.75)  1.56 (0.72)  2.00 (1.13)  1.67 (0.89)  1.57 (0.89)  2.28 (1.21)’  1.73 (0.80)  1.57 (0.82)  1.76 (0.97)  1.58 (0.79)’  1.67 (0.94)  1.69 (0.77)  1.79 (1.04)  1.60 (0.83)  2.02 (0.98)  1.61 (0.86)  1.80 (0.96)  1.58 (0.84)  1.82 (0.98)  1.62 (0.79)  1.66 (0.92)  1.92 (1.14)  1.81 (0.98)  1.48 (0.67)  1.60 (0.86)  1.81 (1.14)  1.71 (1.02)  1.88 (0.93)  2.16 (0.85)  1.74 (0.97)  1.54 (0.82)  1.68 (0.86)  1.62 (0.86)  1.74 (0.92)  1.69 (0.95)  1.57 (0.82)  1.90 (0.93)  1.74 (0.99) | 2.47 (1.27)  2.60 (1.29)  3.09 (1.51)  2.61 (1.29)  2.55 (1.29)  2.64 (1.35)  3.00 (1.50)  2.73 (1.21)  2.55 (1.34)  2.78 (1.33)  2.30 (1.10)  2.50 (1.26)  2.53 (1.29)  2.36 (1.07)  2.86 (1.39)  2.52 (1.20)  2.89 (1.09)  2.41 (1.27)  2.32 (1.10)  2.44 (1.48)  2.71 (1.31)  2.36 (1.21)  3.28 (1.32)  2.55 (1.29)  2.57 (1.29)  3.31 (1.34)  2.27 (1.36)  2.68 (1.27)  2.50 (1.27)  2.56 (1.25)  2.55 (1.36)  2.19 (1.12)  2.76 (1.37)  2.49 (1.27)  2.81 (1.14)  2.57 (1.29)  2.50 (1.27)  2.55 (1.29)  2.56 (1.28)  2.36 (1.22)  2.62 (1.31)  3.00 (1.38)  2.69 (1.27)  2.32 (1.12)  2.53 (1.26)  2.53 (1.46)  2.50 (1.19)  2.37 (1.26)  2.72 (1.28)  2.76 (1.31)  2.51 (1.30)  2.45 (1.22)  2.62 (1.29)  2.47 (1.27)  2.29 (1.27)  2.43 (1.28)  2.79 (1.22)  2.75 (1.30) |
