# Supplementary material for: Chatbot -assisted self-assessment (CASA): Co-designing an AI -powered behaviour change intervention for ethnic minorities
Source: PLOS Digit Health. 2025 Feb 13;4(2):e0000724. doi: 10.1371/journal.pdig.0000724 (PMC11824973; doi:10.1371/journal.pdig.0000724)
Supplement: S2 Table — (DOCX) [file pdig.0000724.s003.docx]

**S2 Table. Means and standard deviations for comfort disclosing information to chatbots by ethnicity.**

| **Ethnicity [N (%)]** | **Comfort disclosing sensitive information [mean; SD]** | **Comfort disclosing demographic information**  **[mean; SD]** | **Comfort disclosing personally identifiable information**  **[mean; SD]** |
| --- | --- | --- | --- |
| White [357 (27.7)] | 2.22 (0.99) | 1.64 (0.98) | 2.62 (1.35) |
| Mixed [108 (8.40)] | 2.21 (0.78) | 1.69 (0.72) | 2.47 (1.22) |
| Asian [360 (28.0)] | 2.33 (0.96) | 1.73 (0.89) | 2.60 (1.25) |
| Black [327 (25.4)] | 2.23 (0.96) | 1.57 (0.79) | 2.40 (1.27) |
| Other [121 (9.40)] | 2.34 (0.98) | 1.65 (0.89) | 2.76 (1.32) |

*5-point Likert scale*
